# Effects of horticultural therapy on elderly’ health: protocol of a randomized controlled trial

**DOI:** 10.1186/s12877-017-0588-z

**Published:** 2017-08-29

**Authors:** Hui Yu Chan, Roger Chun-Man Ho, Rathi Mahendran, Kheng Siang Ng, Wilson Wai-San Tam, Iris Rawtaer, Chay Hoon Tan, Anis Larbi, Lei Feng, Angelia Sia, Maxel Kian-Wee Ng, Goh Lee Gan, Ee Heok Kua

**Affiliations:** 1 Department of Psychological Medicine, Yong Loo Lin School of Medicine, National University of Singapore, Singapore, Singapore; 2 Department of Psychological Medicine, National University Hospital, Singapore, Singapore; 3 Alice Lee Centre for Nursing Studies, Yong Loo Lin School of. Medicine, National University of Singapore, Singapore, Singapore; 4 Department of Pharmacology, Yong Loo Lin School of Medicine, National University of Singapore, Singapore, Singapore; 5 Singapore Immunology Network, Agency for Science, Technology and Research, Singapore, Singapore; 6 Centre for Urban Greenery and Ecology Research, National Parks Board, Singapore, Singapore; 7 Horticulture & Community Gardening Division, National Parks Board, Singapore, Singapore; 8 Division of Family Medicine, National University Hospital, Singapore, Singapore

**Keywords:** Horticultural therapy, Gardening, Elderly, Mental health, Cognitive functioning, Physical health, Randomized controlled trial

## Abstract

**Background:**

Due to a rapidly ageing population in the world, it is increasingly pertinent to promote successful ageing strategies which are cost-effective, easily accessible, and more likely to be acceptable to the elderly. Past research associates exposure to natural environments and horticultural therapy (HT) with positive psychological, social and physical health benefits. This Randomized Controlled Trial (RCT) is designed to evaluate the efficacy of HT in promoting Asian elderly’ mental health, cognitive functioning and physical health.

**Methods/design:**

70 elderly participants aged 60 to 85 years old will be randomized to participate in either the active horticultural therapy group or be in the waitlist control. Sessions will be weekly for 12 weeks, and monthly for 3 months. Mental health will be assessed through self-reports of depressive and anxiety symptomatology, life satisfaction, social connectedness and psychological well-being, collaborated with immunological markers. Outcome measures of cognitive functioning and physical health include neuropsychological tests of cognitive function and basic health screening. Outcomes will be assessed at baseline, 3 months and 6 months post-intervention.

**Discussion:**

This RCT comprehensively investigates the efficacy of a non-invasive intervention, HT, in enhancing mental health, cognitive functioning and physical health. The results have tremendous potential for supporting future successful ageing programs and applicability to larger populations.

**Trial registration:**

ClinicalTrials.gov NCT02495194. Trial registration date: July 13, 2015. Retrospectively registered.

## Background

Worldwide, one in every nine people is aged 60 years and above, and by 2050 the proportion is expected to increase to one in every 5 people [1]. In Singapore, there was a 2.2% increase in the proportion of older persons aged 65 years and above in the recent 5 years [2]. This population has greater mental health risks due to perceived social isolation [3]. Amongst the older adults, gardening is a popular leisure-time activity and exposure to nature has been found to increase positive affect and reduction in anger in individuals, as opposed to those in the urban environment [4]. This may be due to the restorative effects of nature, such as recovery of directed attention fatigue and stress mitigation [5]. Therefore in recent years, there is burgeoning research on therapies which tap on the nature’s benefits on human health and one of which is horticulture.

Definitions of horticultural activities in literature vary in scope from a broad perspective encompassing all forms of exposure to nature and plants, to a more narrow focus on active engagement with plants. Defined broadly, horticultural activities have been shown to reduce depression severity and rumination in clinically depressed patients as the garden environment offers a respite from the urban settings and fascination to them [6]. Notably, besides reducing negative mood (i.e. tension, depression, fatigue, anger and confusion), horticulture also brings about an increase in positive dimension (i.e. vigor/energy) [7]. Similarly, a 7-week indoor horticulture activity program was found to improve participants’ psychological well-being, as compared to the waitlist control group [8]. Hence, horticultural activities may be a promising intervention in improving mental health.

An investigation on the efficacy of horticultural activities in improving mental health, cognitive functioning and physical health is merited for its wider implications. If proven to be effective, this innovative and cost-effective intervention could be a nation-wide strategy in health promotion. It has been stated that the healthcare system alone may be limited in promoting public health [9] and that there is a need for public health strategies to focus on the socio-ecological aspects of health [10]. Therefore, urban parks, which are easily accessible for community-dwelling individuals, are an ideal resource to support the promotion of these aspects of well-being.

Because the present study is a goal-oriented intervention which seeks to enhance the mental, cognitive and physical health of the elderly participants, the definition put forth is adopted:

“Horticultural therapy (HT) is a professionally conducted client-centered treatment modality that utilizes horticulture activities to meet specific therapeutic or rehabilitative goals of its participants. The focus is to maximize social, cognitive, physical and/or psychological functioning and/or to enhance general health and wellness (p. 5).” [11].

Enhanced with the environmental benefits offered by the nature, HT may be a promising intervention in promoting public health and critical in prevention work. However, there is currently a paucity of research on the effectiveness of HT in a multi-ethnic Singaporean population as most studies were conducted either on western societies [6, 12, 13] or Japan and Korea [14]. Due to unique social and cultural specificities, it is inappropriate to assume that the findings could be generalized to our local population.

Furthermore, there are limitations in the existing research. Most of the other studies either have small sample sizes [15–17], short intervention duration [8, 18], or employed the sole use of questionnaires without collaborating evidence from biological data. In addition, a systematic review found that the randomized controlled trials (RCT) conducted thus far have poor methodological and reporting quality [19]. Further research is therefore needed to determine the effectiveness of HT in improving the health of the elderly in the multi-ethnic Singaporean community.

Therefore the aims of this study are to investigate the effectiveness of HT in improving the mental, cognitive and physical health of the community-dwelling older adults using a RCT design. Based on the existing literature, it was hypothesized that, as compared to the waitlist control, participants in the active treatment group will 1) have better mental health, 3) have greater improvement of cognitive functioning and 3) have better physical health. In order to have collaborative evidence with measures of psychological health, the immunology of participants will be investigated via blood-based biomarkers. Notably, past research has found that depressive symptomatology was associated with elevated levels of pro-inflammatory cytokines [20] and lower levels of trophic factor [21].

## Methods

All procedures involved in this trial will be conducted in compliance with the Helsinki declaration and Singapore Guidelines for Good Clinical Practice. The National University of Singapore Institutional Review Board (NUS IRB-Reference Code: B-15-016) gave ethical approval. This paper describes, according to SPIRIT guidelines, the trial design, setting, intervention and procedures that will be undertaken.

### Trial design

This interventional study will be a RCT comparing participation in a HT program to being in a waitlist control. The intervention will lasts for 6 months with 1-h HT sessions during the first 3 months followed by monthly sessions the next 3 months. Outcome assessments will be conducted at baseline, 3 months and 6 months. The 3-months follow-up intervals were decided based on a study by Gonzalez et al. which found that participants’ depressive symptoms declined after 3 months and these improvements were maintained in the final 6-month follow-up [6].

### Study participants

Seventy elderly will be recruited from a research center in the western district of Singapore. All interested and potentially eligible participants will be screened for cognitive status and other eligibility criteria.

English and/or Mandarin-speaking community-dwelling participants aged between 60 to 85 years and have a minimum score of 22 and above on the Montreal Cognitive Assessment (MoCA) will be enrolled into the study. The cut-off score of 22 on MoCA is to select participants who have intact cognitive impairment [22]. However, older adults will be excluded from the study if they have 1) history of severe psychiatric conditions e.g. schizophrenia, bipolar disorder; 2) dementia; 3) significant visual or hearing impairment; 4) marked upper and lower limb motor difficulties, which may affect their ability to participate in the study; 5) are currently suffering from or have history of severe medical conditions e.g. cancer, stroke, Parkinson disease; or 6) are participating in another therapy at the same time.

### Recruitment and baseline procedures

**Fig. 1:**
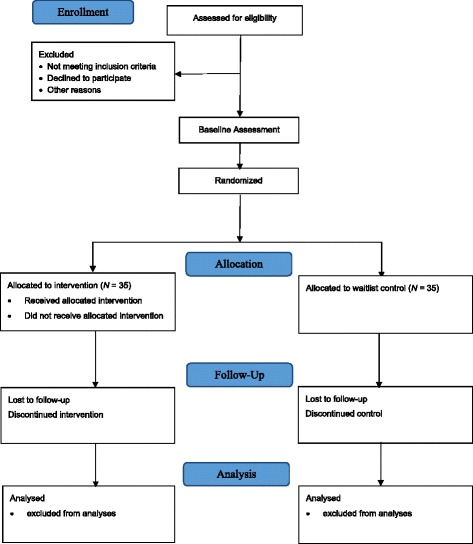
Flow diagram of study protocol

The flow of participants from recruitment to end of study is shown in Fig. 1. Potential participants who may fulfil the inclusion criteria will be invited to participate in the study. They will be contacted either through an invitation phone call or when they are at research centre for activities. The study will be explained in detail to potential participants with the participant information sheet (English and Mandarin versions available) before their written informed consent is obtained. They will be screened by trained research staff using MoCA and demographic questionnaire. Participants who meet the entry criteria will be enrolled into the study.

Following successful screening, enrolled participants will proceed with the rest of the baseline assessments. The participants will be subsequently randomised into either the active treatment group or waitlist control. Utilizing the Random Allocation Software version 2.0 [23], randomization will be stratified by gender by the study-coordinator.

### Intervention

The HT intervention is delivered in 1-h sessions, weekly in the first 3 months, followed by monthly sessions for the next 3 months (see Table 1). The extension of monthly sessions for 3 months is to determine sustainability and longer-term changes. Conducted by a trained practitioner and volunteer facilitators, the intervention is designed to cultivate an interest in gardening and promote relaxation. The participants will be assigned into groups of 7 with a facilitator for enhanced personal engagement. The participants will be with the same group members and facilitator throughout the intervention in order to build rapport and social connectedness. The facilitators are volunteers with the National Parks Board, who were trained with some basic knowledge of horticultural skills such as plant propagation, plant identification, flower arranging, houseplant care, and horticulture crafts. The essential element of the HT program is to communicate to the participants that plants are living things that must be given care. This caring extends the emotional growth of the participants.

**Table 1:** Planned activity sessions for the 6 months of intervention

Session	Topic	Activity	Venue
1	Introduction	1. Familiarisation with research centre 2. Group formation 7/group 3. Indoor Gardening basics	Research Centre
2	Introduction	1. Garden familiarisation 2. Vegetables growing brief 3. Sowing vegetables seedlings	Chinese Garden
3	Wetland Walk	1. Park amenities familiarization 2. Interpretive walk 3. Reflection	Sungei Buloh Reserve
4	Introduction	1. Briefing for vegetables maintenance 2. Weeding and fertilizing vegetables plot	Chinese Garden
5	Nurturing	1. Briefing on pressed flowers 2. Material preparation 3. Make pressed flowers card	Research Centre
6	Nurturing	1. Garden maintenance briefing 2. Weeding, pruning, mulching garden/vegetables	Chinese Garden
7	Colour Walk	1. Park amenities familiarization 2. Interpretive walk 3. Reflection	Singapore Botanical Gardens
8	Nurturing	1. Vegetables maintenance 2. Compost making brief 3. Make compost	Chinese Garden
9	Harvest and Cook	1. Harvest vegetables 2. Hands on preparation for food 3. Sharing of cooked vegetables	Research Centre
10	Harvest and Cook	1. Seed sowing 2. Herbal plant brief 3. Herbal plants propagation	Chinese Garden
11	Festive Walk	1. Park amenities familiarization 2. Interpretive walk 3. Reflection	Gardens by the Bay (Flower Dome)
12	Harvest and Cook	1. Community Garden tour 2. Plant care tips for herbs and plants that will be brought home 3. Reflection	Chinese Garden
13	Healing Walk	1. Park amenities familiarization 2. Interpretive walk 3. Reflection	Botanical Garden – Healing Garden
14	Gardening	1. Briefing 2. Create a culinary garden 3. Maintenance tips	Chinese garden – culinary garden creation
15	Nature Walk	1. Park amenities familiarization 2. Interpretive walk 3. Reflection	Gardens by the Bay (Cloud Forest)

Intervention sessions 1 to 12 will be conducted on a weekly basis while sessions 13 to 15 will be conducted once a month

Each session will start with breathing and stretching exercises, before going into the activity (Table 1). The stretching exercises improve the flexibility of the participants, giving them more freedom of movement for the HT activities. In the first 3 months of weekly sessions, the intervention comprises of 3 main components: 1) indoor horticultural activities, 2) park visits and 3) outdoor gardening. The indoor activities will include making pressed flowers, which is an extension of appreciation for nature, indoor gardening to encourage participants to engage in gardening activity at their home and take ownership of their own plants.

Participants will be brought on visits to various urban parks in Singapore such as the Singapore Botanical Gardens, Sungei Buloh Wetland Reserve and Gardens by the Bay. These visit tours will be facilitated by trained park guides who will share their knowledge on the plants and the landscapes with the participants. Participants will also be encouraged to engage themselves actively in the environment by paying attention to the sights and sounds in the nature parks, thereby appreciating the nature and sharpening their focus. Viewing of natural spaces has been found to reduce anger and increase positive affect, due to the restorative effects of nature [4, 5].

For the gardening activities, they will be held fortnightly at the Chinese Garden where participants will be taught to do weeding and seed sowing. Such active participation in the natural environment has been found to improve mood state where participants were less stressed and were invigorated by their experiences [7]. Vegetables such as ladyfinger, milk cabbage, and choy sum will be planted by the participants. They will be educated on the making of fertilizer as well as knowledge on herbal plants. When their vegetable crops are ready for harvest, the participants will be able to prepare a soup dish together to encourage social connectedness amongst them.

Throughout the intervention programme, there will be an emphasis on education and mental stimulation whereby the facilitation is structured in terms of a series of questions and answers. This encourages active discussion amongst the facilitators and participants and social engagement. Longitudinal studies on mental stimulation and socially integrated lifestyle in late life have been found to have beneficial effect on cognition [24]. In closing each session, participants are asked for their reflection and verbal feedback.

The waitlist control will be assessed at the same time-point as the active treatment group but will only receive the same intervention program 6 months later.

### Assessment

Participants will undergo a thorough assessment using well-validated measures of mental health, biological samples analyses and physical health screening administered by trained research assistants and nurses. Most of the proposed measures have been employed in previous research and have good psychometric properties. These measures will have both English and Mandarin versions.

At baseline, socio-demographic data (such as age, gender, education and previous occupation), medical information, family history and lifestyle factors (such as gardening habits and frequency of park visits) will be obtained via a structured questionnaire. All baseline assessments will be completed in the 3 weeks before commencement of intervention.

#### Measures of mental health

Mental health is defined both in terms of psychopathology as well as positive well-being.

1. Depressive and anxiety symptomatology of older adults will be measured by the Zung Self-Rating Depression Scale (SDS) and the Zung Self-Rating Anxiety Scale (SAS).

- The SDS [25] is a 20-item quantitative measurement of symptoms of depression. Participants rate each item regarding how they felt during the week preceding using a 4-point scale that ranges from 1 (*a little of the time*) to 4 (*most of the time*). The higher the total score, the greater the severity of depressive symptoms. Several studies have established the SDS as a reliable and valid instrument for measuring depressive symptoms in Singaporean Chinese [26].
- The SAS [27] will be used to measure anxiety of the participants in the preceding week. It is a 20-item self-report assessment designed to measure anxiety levels, based on cognitive, autonomic, motor and central nervous system symptoms. Each question is scored on a Likert-type scale of 1–4 (*a little of the time*) to (*most of the time*). Some questions are negatively worded to avoid the problem of set response. Overall assessment is done by total score. The total raw scores range from 20 to 80, with higher score indicating a greater severity of anxiety symptoms.

(2) Well-being will be assessed by the following instruments:

- The Ryff Scales of Psychological Well-Being [28] is an 18-item questionnaire which reflects the six areas of psychological well-being: autonomy, environmental mastery, personal growth, positive relations with others, purpose in life, and self-acceptance. Respondents rate statements on a scale of 1 to 6, with 1 indicating strong disagreement and 6 indicating strong agreement.
- In measuring, life satisfaction, the Satisfaction with Life Scale (SWLS) will be administered [29]. SWLS is a 5-item scale designed to measure global cognitive judgments of one’s life satisfaction (not a measure of either positive or negative affect). It is a psychometrically sound measure [30] and has been validated in a geriatric population [29]. Participants indicate how much they agree or disagree with each of the 5 items using a 7-point scale that ranges from 1 (*strongly disagree*) to 7 (*strongly agree*). Total scores range from 5 to 35, with higher scores indicating higher level of life satisfaction.
- The 6-item Friendship Scale [31] will be administered to measure both social isolation and social connectedness. Participants were asked to rate the frequency in which each statement describes them during the past four weeks on a 5-point Likert scale ranging from 0 (*almost always*) to 5 (*not at all*). Total scores range from 0 to 24, with higher scores indicating higher level of social connectedness. The scale was validated with older adults with excellent internal structures, reliability (α = 0.83) and validity [31, 32].
- In order to gather biological evidence to collaborate with the self-report measures of psychological well-being, the immunology status of participants will be investigated via pro-inflammatory cytokines including Interleukin 6 (IL-6), Interleukin-1 beta (IL-1β), C-reactive protein (CRP), Cortisol, Brain-derived neurotrophic factor (BDNF) and Dehydroepiandrosterone sulphate (DHEAS). Venipuncture will be carried out by qualified research nurses at the research centre. Venous blood (10 ml) will be collected in cell preparation vacuum tubes with sodium citrate (BD plastic vacutainer #362761) for collection of Plasma and Peripheral Blood Mononuclear Cells (PBMC). Samples will be delivered and processed within 3 h after the first venepuncture of the morning, which will be performed at room temperature. Once the samples arrive at the laboratory, 100ul of whole blood, for flow cytometry staining, will be aliquoted into Trucount tube. Subsequently, centrifugation of the samples will be performed at 1650 g, for 25 min at room temperature.
  After centrifugation, plasma will be aliquoted into several sterile 15 ml tubes. Subsequently, PBMC will be re-suspended, transferred into a sterile 15 ml tube and topped up with cold sterile Phosphate-buffered Saline (PBS) and 5% Fetal Bovine Serum (FBS). PBMC will be centrifuged at 1500 rpm for 5 min at 4°C. 500,000 PBMCs will be aliquoted into 2 ml Eppendorf tubes, processed and lysed in 600ul of miRVana Lysis buffer for downstream deoxyribonucleic acid/ ribonucleic acid (DNA/RNA) extraction. Remaining PBMCs will then be further processed, re-suspended in 90% FBS and 10% Dimethyl Sulfoxide (DMSO) and stored in cryovials. All samples will be stored at −80°C until required. Biobanking will be in liquid nitrogen.

#### Assessment of cognitive functioning

- A battery of performance-based neurocognitive tests which evaluates across various cognitive domains including attention, learning, memory, visual-spatial skills and executive function will be administered by trained research assistants. This test battery has been adapted in local population with proved sensitivity in picking up differences with relatively small effect size [33, 34]. Clinical Dementia Rating (CDR) [35] is semi-structured, informant-based clinical interview which is rated based on a 5-point scale which characterize six domains of cognitive and functional performance applicable to Alzheimer disease and related dementias: memory, orientation, judgment & problem solving, community affairs, home & hobbies, and personal care. It has demonstrated convergent validity and discriminatory ability in the local population.
- The Montreal Cognitive Assessment (MoCA) [36] will be administered as global measures of cognitive function. MoCA is a brief screening tool for Mild Cognitive Impairment and Alzheimer Disease and was shown to have higher diagnostic accuracy and sensitivity to cognitive decline than the widely used Mini-Mental State Examination [22] and has been validated in the local population [37].
- Rey Auditory-Verbal Learning Test (RAVLT) [38] that evaluates immediate and delayed recall, learning rate, recognition, proactive and retroactive interference, primacy and recency will be administered
- Digit Span Task [39] that assesses attention and verbal working memory will be administered.
- To assess for sustained attention and sequencing, Colour Trails Tests [40] will be conducted.
- In measuring visual-spatial and organizational processing abilities, as well as non-verbal problem-solving skills, Block Design [39] will be administered
- Semantic Verbal Fluency (Animals) which taps on lexical knowledge and semantic memory organization will also be administered.

#### Assessment of physical health

A basic health screening of blood pressure, pulse rate, height and weight will be measured to investigate the effects of the therapy on physical health of the participants.

### Qualitative data

Feedback questionnaire will be conducted to solicit participants’ subjective experience and thereby expanding our understanding of the impact of the HT intervention. The survey will ask participants to identify what was the most helpful part of the class, what suggestions they have for improvement, and whether they had continued gardening and/or visiting the parks. This brief survey will only be administered once during the post-intervention assessment. Participants’ responses will be informally tallied and listed in categories.

### Sample size

For a pilot RCT with continuous outcomes, the recommended total sample size is 70, with 35 in each study arm with relative change in outcome variables within 95% confidence interval [41].

### Data management

Upon enrollment, each participant will be assigned a unique numeric study number so that they can be tracked anonymously throughout the study and only the Principal Investigator will have access to identifiers that can link the data to the individual participant. Consent forms and hardcopy data collection forms will be stored in a locked cabinet at the research centre and access to them is only made available through the Principal Investigator or trial coordinator.

All research data will be entered into electronic database. Two databases will be created: (1) administrative database which is a Microsoft Excel spreadsheet to compile data related to administrative matters and documentation process. They include identification data, administrative data and regulatory data and, (2) research database which includes a compilation of data that will be used for statistical analysis and outcome measures. This will encompass background data and research data. For this research database, data will be entered directly into Statistical Package for Social Sciences (SPSS) version 23.0 for ease of statistical analysis and the SPSS dataset will be duplicated onto Excel Spreadsheet to facilitate tracking of duplicate, missing and invalid values. For both databases, they will be secure and encrypted with password protection on an external hard drive. Access to the hard drive and password will only be available to Principal Investigator, delegated data manager and data entry staff.

To ensure the accuracy of the research data, three levels of data monitoring will be conducted whereby at the first level, the delegated data manager will check the hardcopies of the data collection forms to ensure accurate scoring. At the second level, data will be entered and at the third level, entries will be checked and approved by the delegated data manager.

### Biomarkers analyses and statistical analyses plan

In the analyses of the biomarkers, aliquoted plasma will be used to measure the cytokines and metabolic profiles of the research participants using single-analyte enzyme-linked immunosorbent assay (ELISA). Using targeted approach, selected RNA targets, which may include messenger RNA and micro-RNA, for a subset of the subject will be analysed based on the data obtained from the cytokines and metabolic profiles. Whole bloods aliquoted on Trucount tubes are stained with a series of fluorescence-conjugated antibodies marking the surface receptors of immune cell. Using flow cytometer, part of the phenotyping includes classical markers of senescence, Cluster of differentiation 57 (CD57) and Killer-cell lectin like receptor G1 (KLRG1), exhaustion of T cells, Programmed cell death protein 1 (PD1) and potentially identification of subpopulations of B cells and myeloid cells with a focus on the pro-inflammatory cells. The extent of DNA damage repair will be assessed by flow cytometry as well. Other immuno-assays aiming at identifying markers or predictors of immune status to examine the effects of the interventions may be applied with the bio-banked samples.

In the data analyses procedures, statistical significance level will be set at .05 for all descriptive and statistical analyses. All analyses will be performed using the SPSS. Based on the initial condition assignment and using the last observation carried forward (LOCF) method, intention-to-treat analyses will be conducted. Preliminary analyses will be performed next to assess the influence of demographic factors that (age, gender, education level, marital status, etc.). Where necessary, these variables will be included as covariates or control variables in the analyses. As some attrition is expected between interviews, prior to data analysis, comparisons will be made of retained and non-retained participants, to determine whether there are any systematic differences.

If there are chance imbalances in baseline participants’ characteristics, they will be entered as covariates in the analyses. Repeated Measure Analysis of Variance (ANOVA) will be used to examine the difference of the outcome between the active treatment group and waitlist control group over the 3 time points [42]. If there are imbalances in baseline participants’ characteristics, those variables will be included as covariates in the analyses. Additionally, dependent t-test will be employed to analyse the changes in scores within the active treatment group across the 3 time-points.

Ratios of selected biomarkers will be taken between cortisol, IL-6, CRP and DHEA-S. Additionally, fold changes and percentage changes would be performed. In the case of significant difference between the baseline measurements of prognostic demographic variables, multivariate regression will be performed when examining the differences between the means of the biomarkers for the two study arms. Correlation and/or heatmap analyses will be carried out to examine the relationships between the biomarker levels. On the aggregate biomarker level, principal component analyses (PCA) will be performed to examine the overall effect of the treatment. Mediational analyses employing bootstrapping technique will be employed to explore the mediating variables between the psychometric outcomes and biomarker levels.

### Protocol compliance and deviation/violation

A Note-to-file will be raised in the site investigator file and serious adverse events will be reported to the NUS-IRB.

## Discussion

This RCT study has been designed to test the effectiveness of HT in improving the mental, cognitive and physical health of the older adults. Studies have shown that contact with nature has been associated with positive mood and improved sleep quality [13] and HT which allows for engagement with nature has been suggested to mitigate stress and improve mood state [7]. This study will be conducted on a multi-racial Asian population to provide substantiation if HT can improve mental health, cognitive functioning or physical health. With the objective measures of biomarkers as collaborative evidence, the proposed study will make significant contributions, both to a theoretical understanding of mental and cognitive health and to providing needed information to the healthcare providers coping with an ageing population.

In addition, this study is evaluating not only quantitative data but also qualitative data via feedback questionnaire. This will provide information on the subjective experience of the participants, and to inform areas of improvement for future programs as ultimately our focus will be on the benefits to the individual and the opportunities to continue such a program beyond the duration of the project.

HT may offer a playful and enjoyable experience to the participants, thereby providing an intrinsic motivation and increasing compliance. Hence, older adults would be self-motivated to appreciate nature and be engaged in gardening even after the program ends. As a basic premise of this community-based initiative, we seek to build the capacity of the participants and empower them to take charge of their own health. This change in lifestyle will enhance their physical activity levels, and consequently increasing their health and ability to cope more effectively with the challenges faced in their daily life.

Community-based HT may provide an avenue for older individuals to socialize more and improve their mental health. After retirement, they are more likely to lead a sedentary lifestyle, with only 30% of them reaching the recommended level of leisure-time activity [43] and slightly more than half (58%) in Singapore were sedentary [44]. Especially for those who are living alone, they might experience loneliness due to a lack of social interaction, which could in turn increase their risk for mental health problems [45]. Participation in HT will help build a sense of social connectedness amongst the older individuals. It is noteworthy that the proposed intervention also includes some physical exercise and educational components aiming to motivate the participants to carry on gardening at home. These various components aim to be holistic in delivering its benefits to the participants, as well as to encourage participants to adopt it as a lifestyle change. In addition, different locations will be used to conduct the intervention. Although this may add to the heterogeneity of such interventions, it reflects that such interventions, which are contingent on its environmental aspects, need to be adjusted to the local and cultural context.

The program can be easily translated to older adults’ activities of daily living. Singapore, termed a “Garden City” has more than 350 parks which are close to homes for easy access [46]. Hence, urban parks are inexpensive resources readily available for the older adults to engage in walks and gardening activities. In addition, as part of the HT program, plants are given to participants to tend to at home, encouraging them to incorporate gardening as part of their daily routine.

Such community-based interventions supported by volunteers are important as they have the potential to be sustained. With its clinical implications, this initiative will guide future larger research trials and healthcare providers of the need to support cost-effective HT as a strategy to engage older adults in a more active and healthier lifestyle. In the context of the growing worldwide mental illness burden of disease, HT will go a long way in easing public healthcare, social and economic costs.

### Study progress

The study has completed data collection.

Intervention programme for the waitlist control and data analyses are currently ongoing. Data analyses are estimated to be completed in October 2016.

## Abbreviations

ANOVA: Analysis of Variance
BDNF: Brain-derived neurotrophic factor
CD57: Cluster of differentiation 57
CDR: Clinical Dementia Rating
CRP: C-reactive protein
CRP: C-reactive protein
DHEAS: Dehydroepiandrosterone Sulphate
DMSO: Dimethyl Sulfoxide
DNA/RNA: Deoxyribonucleic acid/Ribonucleic acid
ELISA: Enzyme-Linked Immunosorbent Assay
FBS: Fetal Bovine Serum
HT: Horticultural Therapy
IL-1β: Interleukin-1 beta
IL-6: Interleukin 6
KLRG1: Killer-cell Lectin like Receptor G1
MoCA: Montreal Cognitive Assessment
PBMC: Plasma and Peripheral Blood Mononuclear Cells
PBS: Phosphate-buffered Saline
PCA: Principal Component Analyses
PD1: Programmed Cell Death Protein 1
RAVLT: Rey Auditory-Verbal Learning Test
RCT: Randomized Controlled Trial
SAS: Zung Self-Rating Anxiety Scale
SDS: Zung Self-Rating Depression Scale
SPSS: Statistical Package for Social Sciences
SWLS: Satisfaction with Life Scale
